# Targeted detection of cancer cells during biopsy allows real-time diagnosis of pulmonary nodules

**DOI:** 10.1007/s00259-022-05868-9

**Published:** 2022-07-05

**Authors:** Gregory T. Kennedy, Feredun S. Azari, Elizabeth Bernstein, Bilal Nadeem, Ashley Chang, Alix Segil, Neil Sullivan, Emmanuel Encarnado, Charuhas Desphande, John C. Kucharczuk, Kaela Leonard, Philip S. Low, Silvia Chen, Aline Criton, Sunil Singhal

**Affiliations:** 1 Department of Surgery, University of Pennsylvania Perelman School of Medicine, 3400 Spruce Street, 6 White Building, Philadelphia, PA 19104 USA; 2 Department of Pathology, University of Pennsylvania School of Medicine, Philadelphia, PA USA; 3 Mauna Kea Technologies, Paris, France; 4 Department of Chemistry, Purdue University, West Lafayette, IN USA; 5 Johnson and Johnson, New Brunswick, NJ USA

**Keywords:** Biopsy, Pulmonary nodules, NIR-nCLE

## Abstract

**Background:**

The diagnostic yield of biopsies of solitary pulmonary nodules (SPNs) is low, particularly in sub-solid lesions. We developed a method (NIR-nCLE) to achieve cellular level cancer detection during biopsy by integrating (i) near-infrared (NIR) imaging using a cancer-targeted tracer (pafolacianine), and (ii) a flexible NIR confocal laser endomicroscopy (CLE) system that can fit within a biopsy needle. Our goal was to assess the diagnostic accuracy of NIR-nCLE ex vivo in SPNs.

**Methods:**

Twenty patients with SPNs were preoperatively infused with pafolacianine. Following resection, specimens were inspected to identify the lesion of interest. NIR-nCLE imaging followed by tissue biopsy was performed within the lesion and in normal lung tissue. All imaging sequences (*n* = 115) were scored by 5 blinded raters on the presence of fluorescent cancer cells and compared to diagnoses by a thoracic pathologist.

**Results:**

Most lesions (*n* = 15, 71%) were adenocarcinoma-spectrum malignancies, including 7 ground glass opacities (33%). Mean fluorescence intensity (MFI) by NIR-nCLE for tumor biopsy was 20.6 arbitrary units (A.U.) and mean MFI for normal lung was 6.4 A.U. (*p* < 0.001). Receiver operating characteristic analysis yielded a high area under the curve for MFI (AUC = 0.951). Blinded raters scored the NIR-nCLE sequences on the presence of fluorescent cancer cells with sensitivity and specificity of 98% and 97%, respectively. Overall diagnostic accuracy was 97%. The inter-observer agreement of the five raters was excellent (*κ* = 0.95).

**Conclusions:**

NIR-nCLE allows sensitive and specific detection of cancer cells in SPNs. This technology has far-reaching implications for diagnostic needle biopsies and intraprocedural decision-making.

**Supplementary Information:**

The online version contains supplementary material available at 10.1007/s00259-022-05868-9.

## Introduction

Increasing use of computed tomography scanning in medical practice and lung cancer screening guidelines has made it commonplace to detect pulmonary nodules radiographically suspicious for malignancy [1, 2]. These solitary pulmonary nodules (SPNs)—spherical radiographic opacities less than 3 cm in diameter that are surrounded by normal lung—present a significant clinical challenge, as the majority are benign but a nontrivial portion represent early-stage, curable lung cancers [3]. There is no clinical or radiological feature that definitively identifies malignant SPNs, and SPNs often require endobronchial or transthoracic tissue biopsy to rule out malignancy [2, 4]. For small, peripheral, or sub-solid nodules, the diagnostic yield of biopsies is low, leading to patient anxiety, increased healthcare costs, additional procedures, and prolonged radiographic surveillance [4–6].

To improve the diagnostic performance of biopsy-based diagnosis and staging, a number of techniques have been proposed to achieve real-time in vivo optical detection of cancer at the cellular level during biopsy. These optical technologies, including Raman spectroscopy, reverse contrast optical endomicroscopy, and fluorescence-lifetime imaging microscopy, require analytic time not conducive to real-time intraprocedural decision-making, cannot be reliably interpreted by non-experts, or do not offer resolution at the cellular level [7–10]. Here, we demonstrate a novel, easily interpreted method of detecting cancer at the single cell level during biopsy.

**Fig. 1:**
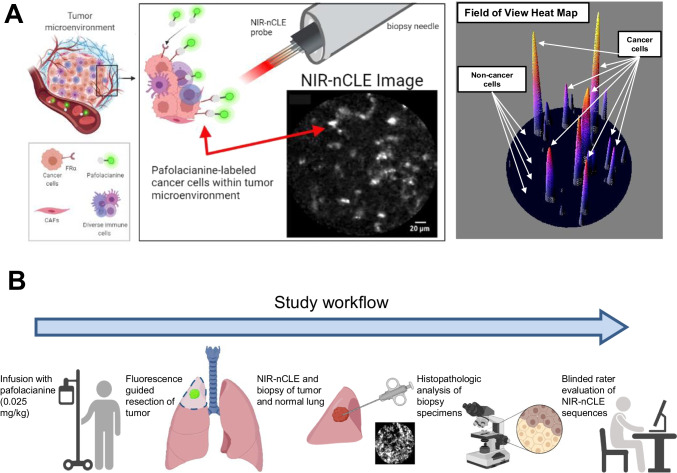
Overview of NIR-nCLE technology and study workflow. **A** Systemically administered pafolacianine localizes to malignant cells overexpressing folate receptor alpha (FRα) within the tumor microenvironment. The NIR-nCLE probe, placed within the lumen of the biopsy needle, detects pafolacianine-labeled cells and thereby locates the optimal site for tissue biopsy. A corresponding NIR heat map of the NIR-nCLE field of view shows fluorescent spikes corresponding to malignant cells. **B** Overview of study workflow

This proposed technology—which we term near-infrared needle-based confocal laser endomicroscopy (NIR-nCLE)—represents the integration of (i) near-infrared (NIR) imaging using a cancer-targeted tracer (pafolacianine) with (ii) an ultra-thin, flexible CLE probe that can fit within the lumen of a biopsy needle to detect optical signal in the NIR range (Fig. 1A). Previous efforts relating to NIR tumor imaging have only focused on macroscopic delineation of tumor from normal tissue during resection [11]. Furthermore, the current state-of-the-art studies in nCLE rely upon reverse contrast imaging that does not specifically detect individual malignant cells [12, 13]. Our approach has been to integrate and synergize these two technologies to solve a clinical challenge.

The tracer pafolacianine is a NIR fluorescent dye (S0456) conjugated to a folate analog, which specifically targets malignant cells overexpressing folate receptor alpha [14]. Intraoperative molecular imaging with pafolacianine has shown efficacy in macroscopic discrimination of tumor from normal tissue during resection of solid tumors, but its ability to localize cancer at the cellular level in vivo has not been evaluated [15, 16].

CLE is an emerging technology in which a low-power laser illuminates a tissue of interest and transmits light reflected from the tissue via a flexible optical fiber bundle to generate high-resolution tissue images [17]. Both probe-based CLE (pCLE) and needle-based CLE (nCLE) permit autofluorescence imaging as well as reverse-contrast imaging of tissues after systemic administration of fluorescein [8, 12, 13, 18]. While promising, these approaches require image interpretation by an experienced observer and do not provide sensitive detection of malignancy at the level of individual cancer cells [3].

In this study, we tested and optimized the technology in ex vivo analysis of pulmonary nodules resected under intraoperative molecular imaging (IMI) guidance using pafolacianine in 20 patients. Our goal was to assess whether NIR-nCLE could achieve rapid, sensitive detection of cancer cells during biopsy procedures with high diagnostic accuracy when compared to formal histopathologic analysis.

## Methods

### Study design

As part of an ongoing study of pafolacianine-guided resection of lung tumors (ClinicalTrials.gov Identifier: NCT02602119), twenty patients with pulmonary nodules concerning for malignancy were enrolled. These patients previously underwent computed tomography (CT) and positron emission tomography (PET) scanning that revealed pulmonary nodules suspicious for malignancy, and they were determined to be good operative candidates by a thoracic surgeon. Exclusion criteria included people under the age of 18, people unable to give informed consent, non-English-speaking people, and patients with prior chest surgery. The study was approved by the University of Pennsylvania Institutional Review Board and all subjects gave written informed consent. Participants received pafolacianine (intravenous, 0.025 mg/kg) 24 h prior to resection. Following resection, the specimens were visually inspected and palpated to identify the lesion of interest on the back table. NIR-nCLE imaging at 3 sites within the lesion and 3 sites in distant, grossly normal lung tissue, and tissue biopsies were taken at the site of imaging (Fig. 1B for study workflow). The ongoing phase 1 trial was not affected by our NIR-nCLE study, since NIR-nCLE imaging was performed on resected specimens after pafolacianine-guided resection.

### Study drug

Pafolacianine (chemical formula C_61_H_63_N_9_Na_4_O_17_S_4_; molecular weight, 1,414.42 Da) is a receptor-targeted tracer consisting of a folate analog conjugated to the NIR fluorescent dye S0456. It has been tested in clinical trials of fluorescence-guided surgery for a number of folate receptor-positive malignancies including non-small cell lung cancer [11, 19, 20], and is now approved by the FDA for guidance of ovarian cancer resection. Pafolacianine maximally excites at a wavelength of 774–776 nm and has a peak emission of 794–796 nm [14].

Pafolacianine (> 96% purity) was obtained via collaboration with Philip Low, PhD (Purdue University, West Lafayette, IN), and On Target Laboratories (West Lafayette, IN). Pafolacianine was synthesized and manufactured at Aptuit in compliance with Good Manufacturing Practices. Pafolacianine was stored at − 20 °C in vials containing 6 mg pafolacianine free acid in 3 mL water.

### NIR-nCLE device

A flexible, ultra-thin NIR-nCLE was developed that was small enough to fit within the lumen of a 19G biopsy needle (Figure S1). The probe contains 10,000 optical fiber bundles with an imaging depth of 55 μm, a lateral resolution of 3.5 μm, and a field of view of 325 μm. The probe was used in conjunction with a commercially available CLE system (CellVizio, Mauna Kea Technologies, Paris, France) that generates an excitation light with a 785 nm laser which is expanded by a beam expander and reflected by a dichroic mirror. Subsequently, a two-dimensional scanning system scans the beam in the two-dimensional plane. After the scanning, the beam is relayed into the back aperture of the objective lens, which couples the excitation light into the proximal end of the optical fiber bundle in the NIR-nCLE probe. Finally, the beam is focused on the sample by a near-infrared miniature objective (at the imaging depth), which also collects the emitted fluorescence from the sample. The fluorescence at longer wavelengths is transmitted along the same path and passed through the dichroic mirror and a bandpass filter (800 to 890 nm). After passing the bandpass filter, the fluorescence is focused by a lens to pass through a pinhole that is placed at the focus of the condenser. The pinhole is used to reject the out-of-focus light. Finally, a photodiode is used to acquire the optical signals and convert them into electrical signals. With the X–Y scanners, the imaging speed can reach 8 to 12 frames per second, which makes imaging in real-time possible. Each frame consists of 320 × 322 pixels.

### Histopathologic and fluorescence analysis of biopsy specimens

All biopsy specimens were imaged with the Odyssey Imaging System (LI-COR Biosciences, Lincoln, NE) or the Iridium Imaging System (Vision Sense, New York, NY). Tissue sections were further analyzed by hematoxylin and eosin staining and fluorescence microscopy (Leica Microsystems, Wetzlar, Germany). Final histopathologic diagnosis was rendered by a board-certified thoracic pathologist.

### Blinded rater training and evaluation of NIR-nCLE video sequences

Five raters, blinded to the histopathology diagnoses of all lesions in the study, underwent a brief (5 min) training session using a standard set of predefined NIR-nCLE sequences and their corresponding pathology diagnosis. The raters had no experience with the NIR-nCLE technology prior to the training session. Following the training session, each rater independently scored a randomized sequence of videos for the presence of various malignancy criteria (presence of pleomorphic cells, clumps of cells, or bright cells) and their diagnosis (malignant/benign) based upon their interpretation of the sequence. Raters immediately scored each 15-s video after watching it once. These ratings were subsequently compared to the final histologic diagnoses associated with each NIR-nCLE sequence. Sensitivity, specificity, positive predictive value, negative predictive value, and accuracy were calculated according to standard definitions. The inter-observer agreement (IOA) and intra-observer reliability (IOR) were calculated with MATLAB R2020b (MathWorks, Natick, MA, USA) using multi-rater Fleiss’ *κ* [21]. The results of the IOA and IOR were interpreted according to the Landis-Koch interpretation system: poor < 0.2, fair 0.21–0.4, moderate 0.41–0.6, substantial 0.61–0.8, and excellent 0.81–1 [22].

### Quantification of fluorescence in NIR-nCLE sequences

The mean fluorescence of each CLE imaging sequence was calculated using MATLAB R2021a (MathWorks, Natick, MA, USA) with dedicated software that finds clusters in histograms allowing the segmentation in NIR-nCLE frames. The average signals in the fluorescent and background areas and standard deviation for each acquisition were calculated. The average tumor fluorescent signal from each NIR-nCLE sequence of the tumor was normalized by the average fluorescent signal from each NIR-nCLE sequence of the normal lung from the same subject by dividing the average tumor fluorescent signal by the average normal tissue fluorescent signal (background) to estimate the tumor-to-background ratio (TBR). The interquartile ranges (IQR) were also calculated.

### Statistical analysis

Statistical comparison of fluorescence intensity values was conducted by paired Student’s *t*-test unless otherwise noted. Receiver operating characteristic (ROC) analysis was conducted using SPSS statistical package V.25.0 to examine the relationship between mean fluorescence intensity values and histopathologic diagnosis. A *p*-value less than 0.05 was considered statistically significant. The sensitivity, specificity, positive predictive value, negative predictive value, accuracy, and the 95% confidence interval (95% CI) were calculated using the standard definitions and software from the SPSS statistical package V.25.0 (IBM Corporation).

## Results

### Patient and lesion characteristics

**Fig. 2:**
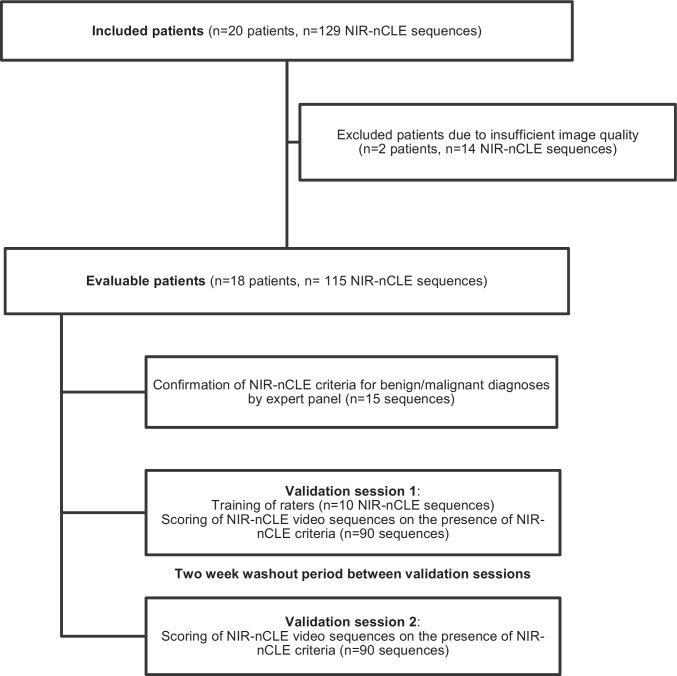
Study flow diagram

Between January and August 2021, 20 patients scheduled for pafolacianine-guided resection of lung lesions suspicious for malignancy were enrolled (Fig. 2 for study flow diagram). Two patients were excluded from the study due to inadequate NIR-nCLE image quality resulting from failed calibration of the NIR-nCLE device. Within the final study cohort (Table 1), patients tended to be female (*n* = 12, 67%), middle-aged (mean age: 62.7 years), and former smokers (*n* = 14, mean pack years = 27.3). There were 18 patients with 21 total lesions, as one patient with metastatic osteosarcoma had 4 metastatic lung lesions resected. Mean lesion size was 2.1 cm (IQR: 1.4–2.2 cm) and mean depth from the pleural surface was 0.5 cm. Most lesions were FDG-avid by preoperative PET scan (mean SUV = 3.6), and 7 (39%) lesions were sub-solid ground glass opacities as determined by a board-certified thoracic radiologist. Mean consolidation-to-tumor ratio (CTR) for GGOs was 0.48 (IQR: 0.38–0.59). Mean standardized uptake value (SUV) of GGOs was 1.9 (IQR: 1.3–2.7). There were no pure GGOs in our study cohort. By final histopathologic analysis, the majority of lesions (*n* = 15, 71%) were adenocarcinoma-spectrum malignancies. The other lesions in the study were metastatic lesions (*n* = 4, 19%), pleomorphic carcinoma (*n* = 1, 5%), or squamous cell carcinoma (*n* = 1, 5%).

**Table 1:** Patient and lesion characteristics

	Number (%) or mean [IQR]
Patient characteristics	
Sex	
Male	6 (33%)
Female	12 (67%)
Age	62.6 [59.3–69.8]
Race	
White	13 (72%)
Asian	3 (17%)
Black	2 (11%)
Former smoker	14 (78%)
Pack years	27.2 [15–37]
Lesion characteristics	
Size of lesion (cm)	2.1 [1.4–2.2]
Depth of lesion (cm)	0.5 [0–0.8]
PET SUV	3.6 [1.8–3.1]
GGO	7 (33%)
Tumor location	
RUL	4 (19%)
RML	2 (10%)
RLL	9 (43%)
LUL	3 (14%)
LLL	3 (14%)
Final pathology	
Invasive adenocarcinoma	13 (62%)
Metastatic osteosarcoma	4 (19%)
Adenocarcinoma in situ	1 (5%)
Minimally invasive adenocarcinoma	1 (5%)
Squamous cell carcinoma	1 (5%)
Pleomorphic carcinoma	1 (5%)
Tumor differentiation	
Well differentiated	4 (21%)
Moderately differentiated	8 (38%)
Poorly differentiated	2 (10%)
Not reported	7 (33%)
Final T stage	
Tis	1 (5%)
Tmi	1 (5%)
T1	8 (38%)
T2	4 (19%)
T3	3 (14%)
n/a	4 (19%)

### NIR-nCLE discriminates cancer from normal tissue in human resection specimens

**Fig. 3:**
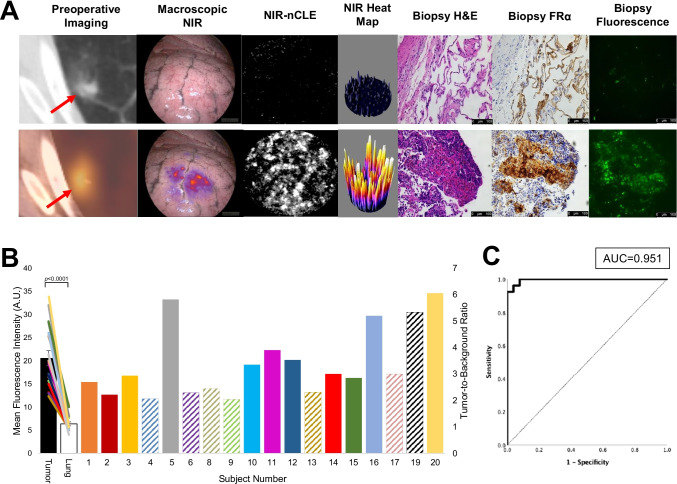
NIR-nCLE identifies pafolacianine-labeled fluorescent tumor cells and distinguishes tumor tissue from normal lung. **A** The leftmost column shows representative preoperative CT and PET imaging from a patient with a sub-solid pulmonary lesion in the study. The subsequent column shows white light and NIR images taken during surgery. The tumor cannot be identified with white light, but is visible using a NIR camera. The following two columns show NIR-nCLE images and corresponding field of view heat maps taken during biopsy of normal lung (top row) and tumor tissue (bottom row). Fluorescently labeled cancer cells are clearly labeled in tumor biopsy, which was confirmed on subsequent hematoxylin and eosin staining (rightmost column). **B** Fluorescence analysis of NIR-nCLE sequences. Black and white bars represent the mean fluorescence intensity (MFI) of tumor and normal lung biopsy sequences, respectively, averaged across all patients. Standard error bars are shown, and the colored lines represent the MFI of tumor and normal lung for individual study participants. The colored bars represent the tumor-to-background ratios of individual subjects and colors correspond to those of lines depicting MFI. Hashed lines indicate ground glass opacities (GGOs). **C** Receiver operating characteristic (ROC) analysis showed that high area under the curve (AUC) was obtained for MFI (AUC = 0.951, 95% CI: 0.909–0.999)

To determine whether NIR-nCLE can differentiate tumor from normal lung in humans, we conducted NIR-nCLE-guided biopsy of tumor and normal lung tissue in resection specimens of patients undergoing pafolacianine-guided lung cancer resection as part of an ongoing phase 1 clinical trial (Fig. 3A for a representative patient). Mean fluorescence intensity (MFI) by NIR-nCLE for tumor tissue was 20.6 arbitrary units ([A.U.], IQR: 15.7–26.0 A.U.) and mean MFI for normal lung was 6.4 A.U. (IQR: 5.1–7/7 A.U., *p* < 0.0001). TBR was greater than 2.0 for all lesions in the study (Fig. 3B), indicating excellent discrimination between tumor and normal tissue. ROC analysis showed that a high area under the curve (AUC) was obtained for MFI (Fig. 3C, AUC = 0.951, 95% CI: 0.909–0.999).

When the analysis was restricted to the GGOs in the study, the trends seen in the entire cohort remained consistent. Mean MFI for GGO lesions was 17.7 A.U. (IQR: 14.3–20.7 A.U.) as compared to 6.8 A.U. (IQR: 5.5–7.8 A.U.) for surrounding normal lung tissue (*p* = 0.001). TBRs for GGOs ranged from 2.1 to 5.8.

### Optimization of NIR-nCLE for discrimination between normal lung and tumor tissue by fluorescent signal thresholding

**Fig. 4:**
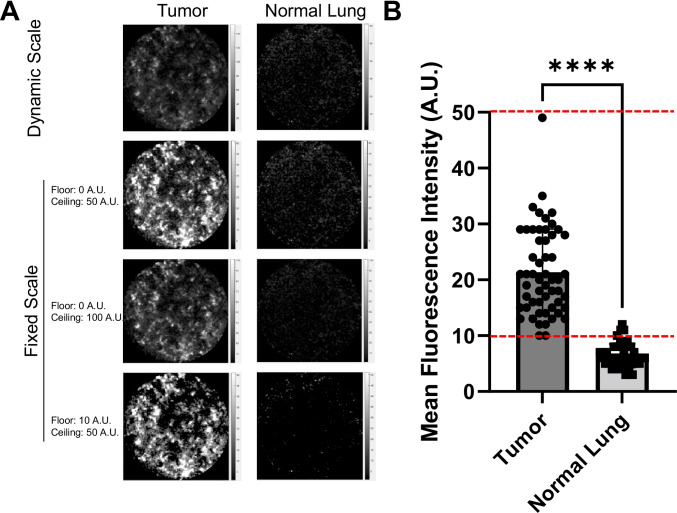
Optimization of NIR-nCLE fluorescence scale for detection of malignant cells. **A** Representative NIR-nCLE images of paired tumor and normal lung tissue when viewed with a dynamic fluorescence scale adjusted to the highest MFI in the field of view (top row) or with a fluorescence scale fixed on predefined fluorescence intensities (bottom three rows). **B** Mean MFI of NIR-nCLE sequences corresponding to tumor biopsies as compared to normal lung biopsies. Columns show mean values with standard error bars, and each point represents an individual NIR-nCLE sequence. Dashed lines indicate the final fixed fluorescence scale used in the study. ****p < 0.0001

To optimize the NIR-nCLE device for detection of pafolacianine-labeled cancer cells and discrimination between tumor tissue and normal tissue, we trialed several different fluorescent scales during display of the NIR-nCLE sequences. Initial sequences were displayed with a dynamic scale, adjusted to the highest fluorescent intensity value in a single field. We found that this strategy obscured the difference between tumor and normal tissue in some cases due to very bright individual cells that artificially dampened the signal of surrounding cells (Fig. 4A for representative case). Based upon MFI analysis of all NIR-nCLE sequences in the study and comparison to final histopathologic diagnosis (Fig. 4B), we trialed three fixed scales for display of NIR-nCLE sequences: 0–50 A.U., 0–100 A.U., and 10–50 A.U. We ultimately found that a fixed scale of 10–50 A.U. provided the greatest contrast between malignant and non-malignant cells while minimizing background fluorescence (Fig. 4A). This scale was used for all subsequent NIR-nCLE imaging and analysis of NIR-nCLE sequences by blinded raters.

### Determination of NIR-nCLE criteria for malignancy

**Fig. 5:**
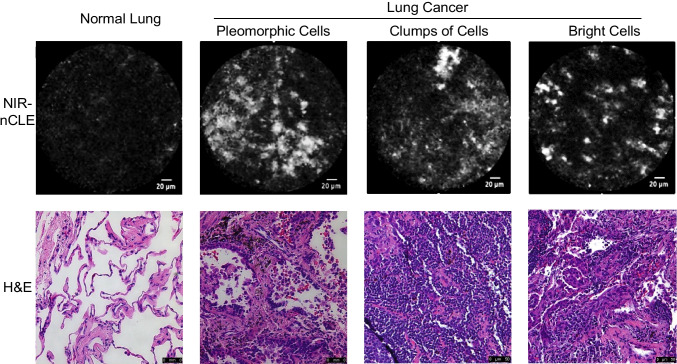
Real-time NIR-nCLE imaging of normal lung and different lung tumors, demonstrating the two previously reported nCLE malignancy criteria (pleomorphic cells and cell clumps) as well as a novel criterion (bright cells), enabled by the tumor-targeted imaging approach used in NIR-nCLE. Hematoxylin and eosin staining of the corresponding biopsy specimens is shown in the bottom row

To determine NIR-nCLE image criteria that were suggestive of a final diagnosis of malignancy, 15 NIR-nCLE sequences (8 from tumor tissue, 7 from normal lung) were reviewed by an expert panel (three thoracic surgeons, one thoracic pathologist, and two CLE researchers) and compared to the corresponding diagnosis on histopathologic analysis. The panel identified three NIR-nCLE criteria for lung cancer as opposed to normal lung tissue: enlarged, pleomorphic cells, clumps of cells, and presence of bright cells (Fig. 5). The former two criteria were identified based upon image characteristics in prior studies of CLE for diagnosis of lung cancer [12], and the third criteria is novel. The NIR-nCLE sequences used for confirmation of the identified airway/lung parenchyma criteria were excluded from the training and validation sessions in order to prevent selection bias.

### Diagnostic performance of NIR-nCLE

To determine the diagnostic performance of NIR-nCLE, five blinded raters reviewed 90 NIR-nCLE sequences corresponding to 39 tissue sites (18 normal lung and 21 malignant tumors, given that 1 patient had 4 lesions resected). When analyzed by individual NIR-nCLE sequence, the blinded raters scored the NIR-nCLE videos on the presence of malignancy with an overall sensitivity and specificity of 86% (95% CI: 81–94%) and 99% (95% CI: 93–100%), respectively (Table 2). Positive predictive value was 99% (95% CI: 93–100%) and negative predictive value was 83% (95% CI: 74–91%). Overall diagnostic accuracy when analyzed on a per sequence basis was 91% (95% CI: 87–95%).

**Table 2:** Diagnostic performance of NIR-nCLE across two validation sessions

First validation session				
Per CLE sequence	Final Diag +	Final Diag −		
nCLE +	46	0	PPV	100%
nCLE −	7	37	NPV	84%
	Sensitivity	Specificity		
	87%	100%		
Per lesion	Final Diag +	Final Diag −		
nCLE +	20	0	PPV	100%
nCLE −	1	18	NPV	95%
	Sensitivity	Specificity		
	95%	100%		
Second validation session				
Per CLE sequence	Final Diag +	Final Diag −		
nCLE +	45	1	PPV	98%
nCLE −	8	36	NPV	82%
	Sensitivity	Specificity		
	85%	98%		
Per lesion	Final Diag +	Final Diag −		
nCLE +	21	1	PPV	95%
nCLE −	0	17	NPV	100%
	Sensitivity	Specificity		
	100%	94%		

When analyzed on a per lesion basis, the blinded raters scored the NIR-nCLE videos on the presence of malignancy with an overall sensitivity and specificity of 98% (95% CI: 87–100%) and 97% (95% CI: 85–100%), respectively. Positive predictive value was 98% (95% CI: 86–100%) and negative predictive value was 98% (95% CI: 86–100%). Overall diagnostic accuracy when analyzed on a per lesion basis was 97% (95% CI: 91–100%).

The overall agreement of the five raters was excellent (Table 3, IOA *κ* = 0.95, 95% CI 0.92–0.98). When ratings were stratified by individual malignancy criteria, the presence of bright cells was recognized with excellent agreement (IOA *κ* = 0.83, 95% CI 0.79–0.87). The presence of pleomorphic cells (*κ* = 0.61, 95% CI 0.60–0.62) or clumps of cells (*κ* = 0.62, 95% CI 0.61–0.63) was recognized with substantial agreement. The calculated IOR by comparing the raters’ performances between the first and second validation session was excellent (*κ* = 0.95 ± 0.02).

**Table 3:** Intra-observer agreement on NIR-nCLE malignancy criteria and overall inter-observer reliability for malignant diagnosis by NIR-nCLE

Criterion	Inter-observer agreement [95% CI]
Pleomorphic cells	0.61 [0.60–0.62]
Clumps of cells	0.62 [0.61–0.63]
Bright cells	0.83 [0.79–0.87]
Final diagnosis	0.95 [0.92–0.98]
Criterion	Intra-observer reliability
Final diagnosis	0.95 ± 0.02

When the analysis was restricted to the GGOs in the study, the trends seen in the entire cohort remained consistent. When analyzed on a per lesion basis, sensitivity of NIR-nCLE for malignancy was 86% (95% CI: 72–100%), specificity was 100% (95% CI: 100–100%), positive predictive value was 100% (95% CI: 100–100%), negative predictive value was 97% (95% CI: 92–100%), and overall accuracy was 98% (95% CI: 89–100%). The calculated IOR remained excellent (*κ* = 0.94 ± 0.04) and the presence of bright cells was recognized with greatest agreement (IOA *κ* = 0.85, 95% CI: 0.83–0.87) as compared to pleomorphic cells (*κ* = 0.59, 95% CI: 0.57–0.61) or clumps of cells (*κ* = 0.74, 95% CI: 0.75–0.79).

## Discussion

In this prospective trial, we demonstrated that NIR-nCLE is feasible for identifying cancer at the cellular level during biopsy, and thereby distinguishing malignant tissue from surrounding normal lung. Importantly, we found that NIR-nCLE delivers easily interpretable images, allowing rapid feedback to pulmonologists and radiologists conducting biopsy procedures. Furthermore, the technology permits rapid, consistent, and highly accurate diagnosis by non-expert observers. Therefore, this study demonstrates the clear clinical promise of NIR-nCLE to improve the diagnostic performance of biopsy-based diagnosis of lung nodules suspicious for malignancy. To the best of our knowledge, this is the first study analyzing NIR-nCLE for the diagnosis of human tumors.

Both CLE and macroscopic NIR pulmonary tumor imaging have been reported previously, but this is the first study to integrate the two technologies and thereby exploit the cancer specificity of the NIR tracer pafolacianine with the cellular-level resolution of CLE. Previous efforts of intra-procedural NIR tumor imaging have focused on the macroscopic identification of tumors and discrimination of the margin between malignant and normal tissue [23, 24]. Our group and others have shown efficacy of this technique in detecting synchronous lesions not identified on preoperative imaging, localizing non-palpable, visually occult lesions, or identifying tumor-positive margins [25–27]. However, NIR imaging has never been evaluated for its ability to detect cancer at the cellular level during procedures.

Initial experiences with CLE in the diagnosis of lung cancer centered upon advancing a CLE probe through the working channel of a bronchoscope and scanning the surface of the bronchial wall [28, 29]. This technique was significantly impeded by the limited depth of penetration of the CLE probes, which did not allow visualization of lung cancers deep to the bronchial wall [30]. This limitation was overcome by the miniaturization of CLE probes, allowing them to fit within the lumen of biopsy needles as small as 19 gauge [13]. Needle-based CLE (nCLE) approaches have shown promise in small studies of bronchoscopic biopsy of central and peripheral lung tumors [12, 13]. This work in nCLE imaging has relied upon tissue autofluorescence or reverse-contrast imaging after systemic administration of fluorescein [8, 18]. nCLE techniques can detect clumps of cancer cells or pleomorphic cells, but display only moderate intra-observer reliability and inter-observer agreement relating to diagnostic image interpretation.

We found that direct tumor targeting with the contrast agent pafolacianine yielded high-quality images that can reliably detect cancer cells, even within small, sub-solid lesions. Directly targeting tumor cells with a contrast agent—as opposed to reverse contrast imaging with fluorescein—allowed a novel nCLE malignancy criterion to be studied: the presence of pafolacianine-labeled, bright cells. We found that there was a much higher degree of inter-observer agreement and intra-observer reliability on this criterion, as opposed to previously proposed malignancy criteria such as pleomorphic cells or cell clumping. We were able to further accentuate the difference between NIR-nCLE images of tumor and normal lung by thresholding the sequences with a fixed scale of mean fluorescence intensity. This adjustment may further streamline the clinical translation of NIR-nCLE technology and make the technology more accessible for non-experts.

In the present study, we conducted a subset analysis restricted to the patients with sub-solid SPNs or GGOs. GGOs are the most challenging pulmonary lesions to evaluate during diagnostic biopsy because they are often early-stage adenocarcinoma spectrum lesions that have a soft tissue architecture not easily distinguishable from normal lung parenchyma [31]. GGOs, unlike solid nodules, cannot be seen using fluoroscopy and radial endobronchial ultrasound (r-EBUS), and no technique is available for real-time visualization of GGOs [3, 32, 33]. In all GGOs evaluated in this study, fluorescent cancer cells were identified within the lesion during biopsy. Blinded, non-expert observers were able to accurately and consistently distinguish NIR-nCLE imaging sequences within the GGOs from those obtained in normal lung parenchyma, pointing to the clinical utility of this technology as an adjunct to bronchoscopic or transthoracic biopsy of GGOs.

This study has several limitations. Because NIR-nCLE is not yet approved for investigational use in humans, we analyzed resected tumors and normal lung tissue from patients that underwent pafolacianine infusion prior to surgery. NIR-nCLE will need to be evaluated in vivo during biopsy procedures to assess the impact of respiratory variation, blood circulation, and motion artifact on the technology’s efficacy. Furthermore, there were no benign lesions captured in our study, which may or may not have impacted the false positive rate of NIR-nCLE. Lastly, we used a folate receptor-targeted NIR tracer, pafolacianine, to detect malignant cells, and note that this tracer may not be effective for tumors that do not overexpress folate receptor alpha. However, we anticipate that NIR-nCLE will be able to detect a broad range of NIR tracers and the choice of tracers can be tailored to individual patients based upon the suspected histopathology of the nodule in question.

In summary, we have demonstrated that NIR-nCLE imaging during biopsy of pulmonary nodules is feasible and allows real-time detection of malignant cells at the tip of the biopsy needle. NIR-nCLE delivers easily interpretable images that permit accurate discrimination between tumor and normal tissue by non-expert observers. This proof-of-concept study analyzed pulmonary nodules as a test case, but the results are likely generalizable to other malignancies and the technology holds great promise for improving the yield of biopsy-based cancer diagnosis.

## Supplementary Information

Below is the link to the electronic supplementary material.

Supplementary file1 (DOCX 464 KB)
