# Supplementary material for: Targeted detection of cancer cells during biopsy allows real-time diagnosis of pulmonary nodules
Source: Eur J Nucl Med Mol Imaging. 2022 Jul 5;49(12):4194–204. doi: 10.1007/s00259-022-05868-9 (PMC9525441; doi:10.1007/s00259-022-05868-9)
Supplement: Supplementary file 1 — Supplementary file1 (DOCX 464 KB) [file 259_2022_5868_MOESM1_ESM.docx]

**Supplementary Figures**


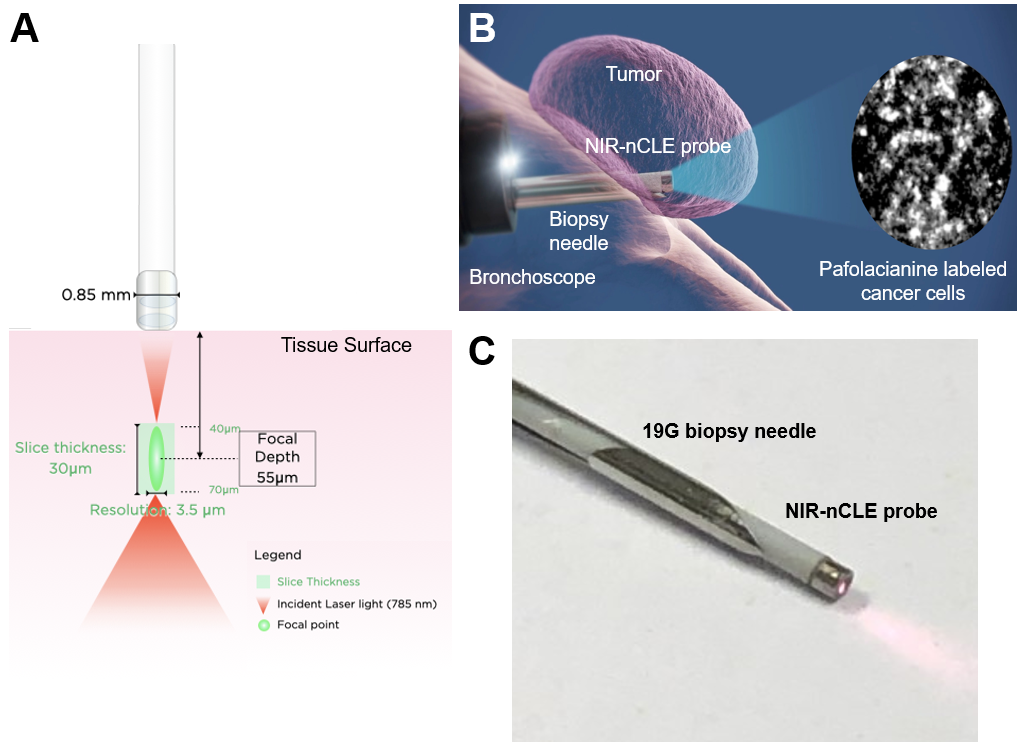


**Figure S1.** **A.** Technical overview of the NIR-nCLE probe tested in the study. **B.** Schematic overview of NIR-nCLE for bronchoscopic biopsy. **C.** Photograph of the NIR-nCLE probe within the lumen of the 19G biopsy needle used in the study.
